# TQT-Covid-19 Study: coping strategies and lessons learned for the future

**DOI:** 10.11606/s1518-8787.2026060S1ap

**Published:** 2026-05-01

**Authors:** Laio Magno, Thais Regis Aranha Rossi, Inês Dourado

**Affiliations:** I Fundação Oswaldo Cruz. Instituto Gonçalo Moniz. Salvador, BA, Brazil; II Universidade do Estado da Bahia. Departamento de Ciências da Vida. Salvador, BA, Brazil; III Universidade Federal da Bahia. Instituto de Saúde Coletiva. Salvador, BA, Brazil

The Covid-19 pandemic constituted a historical landmark, with profound repercussions for health systems, social relations, and the organization of daily life. In May 2023, the World Health Organization declared the end of Covid-19 as a public health emergency of international concern^1^, marking the conclusion of a period characterized by major challenges for health systems and more than three years of intense scientific mobilization focused on knowledge production, expansion of diagnostic testing, vaccine development, implementation of surveillance measures, and provision of clinical care. In this context, several Brazilian initiatives sought to strengthen the health response in territories marked by socioeconomic vulnerabilities.

One of these initiatives is presented in this supplement and is derived from the study "Expanding testing, quarantine, and telemonitoring strategies to address the Covid-19 pandemic in Brazil" (TQT-Covid-19 Study), conducted between July 2022 and July 2023 in primary health care (PHC) units in Salvador, Bahia (BA), and Rio de Janeiro (RJ)^2^. The intervention articulated frameworks from health surveillance, digital health, community mobilization, and the reorganization of care practices, constituting an important contribution to the analysis of the multiple challenges faced by PHC during a health emergency.

The eleven articles comprising this supplement were organized to address the research questions outlined in the study protocol, encompassing the trajectory of the intervention, its structuring axes, and its main results. The supplement begins with analyses focused on community practices and perceptions related to prevention. The study by Amorim et al.^3^ described patterns of adherence to preventive behaviors against Covid-19 in communities in Salvador and Rio de Janeiro, highlighting significant inequalities. Along the same lines, Castanheira et al.^4^ explored factors associated with the use of ineffective medications for Covid-19 prevention, revealing the persistence of misinformation and inequalities in access to scientific information.

In the third article, Vieira et al.^5^ further analyzed care related to chronic conditions in PHC during the pandemic, highlighting disruptions in the follow-up of individuals with hypertension and diabetes, as well as weaknesses in care coordination. These studies contributed to understanding the context in which the TQT strategy was implemented in both cities.

The subsequent articles addressed key components of the intervention. Victor et al.^6^ demonstrated that the ability to comply with home isolation after a Covid-19 diagnosis was strongly associated with housing conditions, particularly household density, highlighting the importance of social determinants in surveillance actions. Subsequently, Cruz et al.^7^ analyzed the perceptions of PHC professionals and managers regarding Covid-19 testing, identifying structural and organizational challenges, such as resource scarcity and weaknesses in coordination between management levels. Complementarily, Zeballos et al.^8^ described community mobilization strategies that expanded access to testing and analyzed surveillance indicators, pointing to the main difficulties in implementing telemonitoring and contact tracing.

Within the dimension of intervention monitoring, Oliveira et al.^9^ examined the TQT-Covid-19 Study proposal and the implementation of follow-up actions for the planned activities, identifying advances in the expansion of testing and limitations in the consolidation of surveillance actions within the primary health care work process. Similarly, Soares et al.^10^ reinforced the potential of self-testing as a complementary strategy to conventional testing, particularly among individuals with higher levels of education and prior familiarity with the device.

The subsequent articles addressed fundamental dimensions of health work during the pandemic. Mendonça et al.^11^ analyzed how the health crisis exacerbated historical inequalities, deepening work precariousness and increasing physical and mental illness among PHC workers. Complementarily, Castanheira et al.^12^ described the impacts of overload, fear, and lack of psychological support on the mental health of health professionals in the context of the pandemic, with particular emphasis on community health workers.

The supplement concludes with the intervention evaluation article, in which Silva et al.^13^ integrated the various dimensions addressed in the previous studies, highlighting important advances in expanding access to testing, while also identifying structural and organizational challenges for the consolidation of surveillance practices in PHC.

Collectively, these articles in this supplement demonstrated that the TQT-Covid-19 Study intervention expanded access to testing, stimulated surveillance practices, and contributed to improving the local response to the pandemic in contexts of high social vulnerability. At the same time, they revealed structural weaknesses in PHC, such as insufficient human resources, territorial inequalities, precarious working conditions, and difficulties in integrating surveillance and health care. These findings reinforced the need for continuous investment in PHC and a revision of primary care policies in Brazil, as well as the qualification of teams, expansion of the use of digital technologies, and strengthening of health communication policies aimed at addressing misinformation and promoting public trust.
