# Supplementary material for: TQT-Covid-19 Study: coping strategies and lessons learned for the future
Source: Rev Saude Publica. 2026 May 1;60(Suppl 1):e2s. doi: 10.11606/s1518-8787.2026060S1ap (PMC13143266; doi:10.11606/s1518-8787.2026060S1ap)
Supplement: Translation

# Translation: Estudo TQT-Covid-19: estratégias de enfrentamento e aprendizados para o futuro

**Authors:** Laio Magno, Thais Regis Aranha Rossi, Inês Dourado

**Affiliations:** I Fundação Oswaldo Cruz. Instituto Gonçalo Moniz. Salvador, BA, Brasil; II Universidade do Estado da Bahia. Departamento de Ciências da Vida. Salvador, BA, Brasil; III Universidade Federal da Bahia. Instituto de Saúde Coletiva. Salvador, BA, Brasil

A pandemia de covid-19 constituiu um marco histórico, com repercussões profundas sobre os sistemas de saúde, as relações sociais e a organização da vida cotidiana. Em maio de 2023, a Organização Mundial da Saúde declarou o fim da covid-19 como emergência de saúde pública de importância internacional^1^, marcando um período de grandes desafios para os sistemas de saúde, com mais de três anos de intensa mobilização científica em torno da produção de conhecimento, ampliação dos testes diagnósticos, desenvolvimento de vacinas, adoção de medidas de vigilância e oferta de cuidados clínicos. Nesse contexto, diversas experiências brasileiras buscaram fortalecer a resposta sanitária em territórios marcados por vulnerabilidades socioeconômicas.

Uma dessas iniciativas é apresentada neste suplemento, derivada do estudo "Ampliação das estratégias de testagem, quarentena e telemonitoramento para o enfrentamento da pandemia de covid-19 no Brasil" (Estudo TQT-Covid-19), desenvolvido entre julho de 2022 e julho de 2023, em unidades da atenção primária à saúde (APS) de Salvador (BA) e do Rio de Janeiro (RJ)^2^. A intervenção articulou referenciais da vigilância em saúde, da saúde digital, da mobilização comunitária e da reorganização das práticas de atenção, constituindo-se importante contribuição para a análise dos múltiplos desafios enfrentados pela APS durante uma emergência sanitária.

Os onze artigos que compõem este suplemento foram organizados de modo a responder às perguntas de investigação do protocolo de pesquisa, contemplar a trajetória da intervenção, seus eixos estruturantes e principais resultados. O suplemento inicia-se com análises voltadas às práticas e às percepções comunitárias relacionadas à prevenção. O estudo de Amorim et al.^3^ descreveu padrões de adesão a comportamentos preventivos da covid-19 em comunidades de Salvador e do Rio de Janeiro, evidenciando desigualdades importantes. Na mesma direção, Castanheira et al.^4^ exploraram os fatores associados ao uso de medicamentos ineficazes para prevenção da covid-19, revelando a persistência da desinformação e de desigualdades no acesso à informação científica.

No terceiro artigo, Vieira et al.^5^ aprofundaram a análise do cuidado com relação a condições crônicas na APS durante a pandemia, evidenciando rupturas no acompanhamento de pessoas com hipertensão e diabetes e fragilidades na coordenação do cuidado. Esses estudos contribuíram para a compreensão do contexto no qual a estratégia TQT foi implementada em ambas as cidades.

Os artigos seguintes abordaram componentes centrais da intervenção. Victor et al.^6^ demonstraram que a possibilidade de cumprir o isolamento domiciliar após o diagnóstico de covid-19 esteve fortemente associada às condições habitacionais, especialmente à densidade domiciliar, ressaltando a importância dos determinantes sociais nas ações de vigilância. Em seguida, Cruz et al.^7^ analisaram a percepção de profissionais e gestores da APS sobre a testagem para covid-19, identificando desafios estruturais e organizacionais, como escassez de recursos e fragilidades na coordenação entre os níveis de gestão. Complementarmente, Zeballos et al.^8^ descreveram as estratégias de mobilização comunitária que ampliaram o acesso à testagem e analisaram indicadores de vigilância, apontando as maiores dificuldades na implementação do telemonitoramento e do rastreamento de contatos.

Na dimensão do monitoramento da intervenção, Oliveira et al.^9^ examinaram a proposta do Estudo TQT-Covid-19 e a implementação das ações de acompanhamento das atividades previstas, identificando avanços na expansão da testagem e limitações na consolidação das ações de vigilância no processo de trabalho da APS. Na mesma direção, Soares et al.^10^ reforçaram o potencial do autoteste como estratégia complementar à testagem convencional, especialmente por indivíduos com maior escolaridade e familiaridade prévia com o dispositivo.

Os artigos seguintes abordaram dimensões fundamentais do trabalho em saúde durante a pandemia. Mendonça et al.^11^ analisaram como a crise sanitária exacerbou desigualdades históricas, aprofundando a precarização do trabalho e ampliando o adoecimento físico e mental dos trabalhadores da APS. De forma complementar, Castanheira et al.^12^ descreveram os impactos da sobrecarga, do medo e da ausência de apoio psicológico na saúde mental dos profissionais de saúde no contexto da pandemia, com destaque para os agentes comunitários de saúde.

O suplemento finaliza com o artigo de avaliação da intervenção, no qual Silva et al.^13^ integraram as diversas dimensões abordadas nos estudos anteriores, evidenciando avanços importantes na ampliação do acesso à testagem, ao mesmo tempo que identificaram desafios estruturais e organizacionais para a consolidação das práticas de vigilância na APS.

Conjuntamente, os artigos deste suplemento demonstraram que a intervenção do Estudo TQT-Covid-19 ampliou o acesso à testagem, estimulou práticas de vigilância e contribuiu para qualificar a resposta local à pandemia em contextos de alta vulnerabilidade social. Ao mesmo tempo, revelaram fragilidades estruturais da APS, como insuficiência de recursos humanos, desigualdades territoriais, precarização do trabalho e dificuldades de integração da vigilância e da atenção à saúde. Esses achados reforçaram a necessidade de investimentos contínuos na APS e de revisão das políticas de atenção básica no Brasil, bem como da qualificação das equipes, da ampliação do uso de tecnologias digitais e do fortalecimento de políticas de comunicação em saúde que enfrentem a desinformação e promovam a confiança da população.
